# Revisiting the need for innovative oral hydration technologies

**DOI:** 10.3389/fnut.2026.1851906

**Published:** 2026-09-23

**Authors:** Genevieve Vine, Simone Persaud, Austin Swiniarski, Eva Rosaasen-Uwiera, Spencer D. Proctor

**Affiliations:** 1 Metabolic and Cardiovascular Diseases Laboratory, Division of Human Nutrition, University of Alberta, Edmonton, AB, Canada; 2 Best Hydrate Inc, Edmonton, AB, Canada; 3 Department of Psychology, University of Alberta, Edmonton, AB, Canada

**Keywords:** dehydration management, electrolyte balance, exercise, hydration formula, oral rehydration solution, oral rehydration therapy

## Abstract

Oral rehydration therapy (ORT) is an important component of fluid management in people with dysregulated fluid intake, renal disorders, and inflammatory gastrointestinal diseases. During intense exercise or periods of compromised intestinal water absorption, regulated intake of an oral rehydration solution (ORS) promotes water uptake and replenishes depleted micronutrients. The primary components of ORS formulation are sodium and glucose, which are co-transported across the apical membrane of ileal and jejunal epithelia to create an osmotic gradient favorable to water uptake. ORS designed for specific conditions are formulated with additional sugars, starches, micronutrients, and proteins to enhance water uptake and counteract solute loss. Despite its efficacy in preventing mortality in children with watery diarrhea, and its demonstratable benefits over intravenous fluid replacement in clinical applications, there is a lack of recent published developments in ORS technology. Recent developments have been driven by the sports nutrition industry, particularly in targeted delivery of carbohydrates. These proprietary developments have potential applications in ORS formulation to improve chronic disease outcomes and enhance acute fluid retention during clinical crises. This review aims to summarize recent literature investigating ORS formulation and application with a focus on chronic disease and exercise rehydration, as well as demonstrate the opportunity for development of new rehydration technologies for both general fluid maintenance and clinical fluid replacement therapy.

## 1 Introduction

Rehydration or fluid replacement is the process of restoring the body's fluids and electrolytes. Total water intake includes drinking water, water in beverages, and water in food. The median water intake from US survey data indicates an adequate daily water intake of 3.7L for men and 2.7L for women aged 19–30 to prevent deleterious effects of dehydration, including metabolic and functional abnormalities (1). Daily fluid requirements are higher in individuals who frequently engage in strenuous exercise, are exposed to significant heat or altitude, or are losing fluid through vomiting or diarrhea (2–4). In the US, 81% of total water is consumed in fluids with the remaining being consumed in food (1). Drinking water is the main source of fluid intake with others being coffee, tea, carbonated drinks, juice, and milk. Metabolic water production also contributes to water gain, with the rate of formation dependent on energy expenditure, metabolic rate, and the substrates being metabolized for energy (5). Approximately 250–350 mL per day for sedentary persons and 500–600 mL/d for physically active persons is generated this way (1).

Oral Rehydration Solutions (ORS) are hydration fluids formulated to promote water uptake and rebalance electrolytes. Hydration is achieved by modifying gastric emptying, intestinal absorption, fluid retention, and renal extraction to maximize water retention (3). The rate of gastric emptying may be the main factor that limits absorption of fluids, being maximized with large gastric volume and inhibited by nutritional content in the proximal small intestine (6, 7). Lower energy density beverages empty faster than higher energy density beverages, suggesting that ideal rehydration is achieved with moderate energy content with electrolytes that facilitate a negative osmotic gradient in the intestinal lumen.

Oral rehydration therapy (ORT) is a dehydration treatment regime in which electrolyte rich iso- or hypo-tonic solutions are administered orally in small amounts throughout the day. The advantages of ORT compared to parenteral or intravenous (IV) hydration are that it is convenient and easy to administer, and can even be self-administered (8). It is non-invasive and typically more affordable (9). The disadvantages are that hydration takes place more slowly when the gastrointestinal tract is damaged, and patients with paralytic ileus or persistent vomiting may be excluded (4). Reviews and meta-analyses have found no clinical difference between oral vs. intravenous fluids, concluding that oral rehydration should be the first-line therapy for mild to moderate dehydration with IV therapy only being used if oral therapy fails (10).

Oral rehydration therapy was widely used for the purpose of preventing losses through the intestinal tract in patients with Vibrio cholera infections, but some of the earliest reports of the effect of hydration on work capacity are from the military (6). The success of military operations has been decided over access to water, particularly in hot and dry climates. Early studies on marching soldiers found that exercising men only drank *ad libitum* about 2/3 of the loss accrued from sweating, and hydration in a volume that matches sweat loss was more beneficial. In 1947 it was found that drinking salty fluid reduced renal water excretion, and flavorants increased *ad libitum* fluid intake (6).

Glucose-containing ORS increases absorption of water and electrolytes and significantly improves survival rates during severe diarrhea (2). ORT was shown to decrease mortality, length of hospitalization, and cost associated with diarrheal illness in children. When glucose-sodium based ORT is given in equal volumes to diarrheal losses, there is a significant reduction in the need for IV fluid therapy and a 10 fold reduction in mortality compared to those on IV therapy (11). However, ORT does not reduce severity of diarrhea in volume or duration. Oral rehydration has been acclaimed as one of the most important therapeutic advances of the 20th century for its effectiveness in treating acute watery diarrhea (12). As of 2007 it was estimated that ORT had prevented 54 million deaths from diarrheal disease (13).

Despite an improved understanding of renal function, gastric physiology, and carbohydrate metabolism, as well as the reported efficacy of ORS over IV therapy, investigations have been limited in the development of optimized hydration solutions for both acute and chronic clinical applications. In contrast to the relatively slow progression of clinical ORS hydration technologies, the sports nutrition field has made rapid advancements in hydration science through improvements in carbohydrate delivery, electrolyte composition, and fluid retention strategies. The goal of this review is to provide a unified perspective by examining the physiological mechanisms governing fluid balance and dehydration, discussing the formulation principles of ORS and exploring their applications in both exercise and clinical populations. By integrating evidence from both medicine and sports nutrition, we emphasize opportunities to translate advances in athletic hydrations to clinical settings in the treatment of patients with chronic and acute fluid balance disorders.

## 2 Methods

Literature searches were conducted to identify studies relevant to oral rehydration in exercise and chronic disease in the last two decades. Searches were performed using the PubMed database on March 24 and May 8 2025. Search terms included those directly relating to oral rehydration solutions (oral, rehydration, solution, therapy, fluid balance, body water), as well as keywords regarding dehydration disorders (dehydration, chronic, treatment, acute, clinical, hypernatremia, polyuria, short bowel syndrome, irritable bowel disease, Crohn's disease, diabetes mellitus, diabetes insipidus, outpatient, ketoacidosis, ketoanemia, intravenous fluid therapy), formulation (osmolality, electrolytes, hypotonic, isotonic, zinc, magnesium, ketone, probiotic, carbohydrate, protein), and athletics (exercise, recovery, endurance, performance). Studies on watery diarrhea, gastroenteritis, and Vibrio cholera infections were excluded. Studies involving animals were only included if exceedingly relevant or significant. Initially, only peer-reviewed articles published in English between January 2005 and May 2025 were considered.

Searches returned 232 entries which were screened for inclusion based on satisfying at least one of the following criteria: (i) Provides a new insight or development into understanding dehydration; (ii) Addresses a specific medical cause of dehydration that is treatable with ORS; (iii) Investigates the addition of novel substrates to increase ORS effectiveness; (iv) Investigates novel applications of ORS technology. One hundred and twenty-two full- text articles were assessed for relevance, and 86 peer-reviewed articles are included in this review.

Additional sources provide fundamental context to the field and exemplify unpublished technological developments. Ten studies published prior to 2005 were included for their foundational or large-scale contributions to dehydration and rehydration technology. Seven non-peer reviewed sources are also cited, including textbooks, and global guidelines. Two additional articles from 2026 were included at the review stage. Nineteen of the 96 selected peer-reviewed articles are clinical trials involving human participants, and their results are summarized in Table 1.

**Table 1:** Literature extraction table summarizing clinical trials from the last 30 years (2005–1995) that demonstrate significant findings with regards to rehydration applications, oral hydration composition, and chronic disease management.

References	Country	Rationale	Aims	Study type	Population	Sample size	Age	Setting	Treatments	Outcomes	Key findings
Boyd-Shiwarski (106)	USA	How does ingestion rate and beverage composition affect hydration?	Examined three beverages with varying ingestion rates and measured hydration.	Cross-over, Placebo-controlled	Healthy adults (male and female)	30	18–45	Clinic	Buoy hydration drops, Nuun sport, bottled H2O (1L)	Urine volume, BHI, fluid balance	Buoy improved hydration markers after 6 h compared to water and Nuun.
Millard-Stafford et al. (108)	USA	Different additives to electrolyte drinks have been explored to increase fluid retention when rehydrating following exercise.	Determined whether electrolytes, carbohydrates, or dipeptides contribute the most to rehydration.	Randomized, double blind, Crossover	Healthy young adults	19	24.7 ± 5.6	Clinic	Deionized H2O, electrolytes only, CHO-electrolytes, alanyl-glutamine-electrolytes	BHI, net fluid balance	Electrolytes alone did not improve BHI compared to water, but the combinations improved fluid retention compared to water.
Maughan et al. (107)	UK	Development of a beverage hydration index for evaluating fluid retention.	Measured the diuretic effect of 13 commercially available beverages on euhydrated males.	RCT	Recreationally active, euhydrated males	72	18–35	Three-site clinical trial	Still H2O, sparkling H2O, Coke, Diet Coke, Powerade, Dioralyte ORS, orange juice, lager, coffee, tea, milk	Cumulative urine mass, BHI (cumulative urine mass of water/beverage)	Full fat milk, skimmed milk, ORS, and Orange juice had higher BHI than water. Highest electrolyte content tended to have the highest BHI.
Seifert et al. (38)	USA	Protein enhances sodium and water absorption in the intestine. Amino acids increase transcellular and paracellular water absorption.	Evaluate rehydration potential of protein added to sports drink after dehydration with exercise.	RCT	Endurance Athletes	13	20–28	Clinic	CHO/electrolyte drink, CHO/ electrolyte/ protein drink, flavored H2O	Serum and urine osmolality, fluid retention	Fluid retention was greater in drink with protein added. Serum and urine osmolalities were greater in protein group than carbohydrate/ electrolyte, which were greater than water group.
Marshall et al. (23)	USA	Water transport and local airway hydration are critical for normal functioning of lungs and airways. Even mild dehydration can have unfavorable effects on multiple organ systems.	Determine effect of exercise and fluid-restriction induced dehydration on pulmonary function. Does systemic or local hydration help reverse the effects?	Repeated-measures, Randomized, Cross over	Healthy non-smoking adults	10	29 ± 8	Clinic	Oral saline, ultrasonic nebulization with isotonic saline	Forced vital capacity, residual volume, functional residual capacity	Dehydration-caused pulmonary impairment were reversed by oral hydration (systemic) but not nebulization (local).
Bhatnagar et al. (53)	India	Observationally, low plasma zinc is associated with worse diarrhea outcomes. Zinc deficiency is common in children from developing countries. Zinc fecal losses increase during diarrhea.	Evaluate the effect of zinc treatment as an adjunct to oral rehydration therapy in children with diarrhea.	Double-blind RCT	Dehydrated male children	287	3–36 months	Three-site hospital trial	Zn, placebo	Stool output, diarrheal output	Zinc reduced total stool output, output per day of diarrhea, risk of continued diarrhea, and proportion of episodes ≥5 days.
Hurt et al. (36)	USA	Compliance is a barrier to receiving ORS in SBS patients undergoing home parenteral nutrition.	Evaluate the taste rating and compliance of two different ORS solutions, one made locally, and the other commercially available.	Double blind RCT	Patients with SBS	19	34–76	Clinic	WHO ORS (245 mOsm/L), DripDrop (215 mOsm/L)	Taste rating 1–10, compliance	Taste rating was 7.3 ± 1.9 for WHO ORS and 7.6 ± 1.6 for DripDrop. This was not statistically significant. The commercial ORS had slightly better compliance, but was much more expensive.
Beaugerie et al. (40)	France	Glutamine is actively absorbed in the small intestine in a similar way to glucose. It is a source of energy for intestinal epithelial cells.	Compare the effects of standard WHO-ORS with polymeric glucose solution + glutamine	Randomized cross over	Patients with high jejunostomy	6	28–78	Hospital	WHO-ORS, glutamine-enriched ORS	8 h net fluid absorption, sodium absorption	8 h fluid absorption was not significantly different; sodium absorption was better in standard WHO-ORS.
Taniguchi et al. (83)	Japan	Preoperative fluids reduces anxiety, dry mouth and hunger following fast. Carbohydrate loading reduces. Postoperative insulin resistance.	Investigate efficacy of ORT for preoperative fluid and electrolyte balance	Prospective observational	Breast surgery patients	20	20–79	Clinic	1L ORS-1 (270 mOsm/L) 1 h pre-operation	Fractional excretion of sodium, EPGF volume	ORT is a safe and effective therapy for preoperative management of fluid and electrolytes.
Ijiri et al. (84)	Japan	Carbohydrate loading before surgery improves preoperative stress and postoperative conditions compared with traditional fasting.	Does preoperative ORS stabilize perioperative circulatory dynamics during cesarean section?	RCT	Elective cesarean patients	61	31.67 (median)	Clinic	ORS, mineral H2O, no fluid	Vasopressor boluses, dose of vasopressors	ORS group required fewer vasopressor boluses than control and required a smaller phenylephrine dose.
Milner et al. (82)	USA	IV fluids are usually used when treating thermal injuries. ORT may have the potential to save lives in resource-poor areas where transport, intensive care, and and definitive surgical care may be delayed.	Does ORT reduce the amount of IV fluids needed? This would suggest an appropriate replacement for IV within 24 h of accident.	Prospective, Case study	Patients with burn wounds 20–40% of the body	3	41 (median)	Clinic	Ceralyte90 rice based ORS (270 mOsm/L)	Urine output, gastric residual	Adequate tissue perfusion with ORT supplementation. Mean 65.2% of IV fluids were replaced successfully with oral fluids.
Shirreffs et al. (47)	UK	Milk has naturally high electrolyte content, carbohydrate similar to sports drinks, and additional protein.	Does milk effectively restore fluid balance after exercise-induced hypohydration?	Randomized cross over	Healthy volunteers	11	24, SD 4	Clinic	Low fat milk, milk plus 20 mmol/L Na, powerade, H2O	Cumulative urine output, net fluid balance	Milk may be an effective post-exercise rehydration drink. Increase in urine output up to 2 h after drinking powerade and water. Cumulative urine output was less for the milk drinks. Net positive or neutral fluid balance was observed in the milk groups for the entire recovery period, but was negative in the other groups.
Nightingale et al. (96)	UK	Patients with end jejunostomy have high losses of fluid and electrolytes. 120 mmol/L sodium solutions are optimal. Glucose polymers allow more glucose to be dissolved in an isotonic solution.	Are sodium capsules as effective as glucose electrolyte solution containing the same NaCl? Does a glucose polymer increase the amount of sodium or energy absorbed?	Randomized cross over	Patients with jejunostomy (residual jejunal length 105–250 cm)	6	26–65 (50.5 mean)	Hospital	Gelatine salt capsules, isotonic glucose electrolyte solution, glucose polymer solution (Maxijul)	Daily stomal output, sodium balance, energy absorption	No patient who took the electrolyte-glucose solution were in negative sodium balance after eight days. The other treatments had at least 1 patient in negative salt balance. Small increase in energy absorption in Maxijul group compared to electrolyte-glucose solution group.
Nishinaka et al. (32)	Japan	Optimizing water and electrolyte absorption with ORS	Assessed the relationship between osmolality and water absorption, as well as electroyte absorption and ion concentration.	Experimental trial	Male Wistar rats	25	9 weeks	Laboratory	Solita-T No. 2, Solita-T No. 3, Aqualight, Pocarisweat, distilled water	Water and electrolyte absorption	Hypotonic ORS significantly promoted net water absorption compared to isotonic solutions. So was net sodium absorption. Sodium absorption was greater in the colon than the small intestine. Water did not contribute to water absorption in the small intestine but was equivalent to hypotonic solutions in the colon. Water induced the most sodium secretion.
Maragkoudaki et al. (100)	Greece	Selected strains of probiotics have been shown to reduce duration and severity of acute diarrhea in children. L reuteri strains have shown to have a beneficial effect on duration and severity of acute gastroenteritis.	To test L. reuteri DSM 1738, which already shows promise in children with acute diarrhea, in combination with ORS as a commination treatment for infant and toddler diarrhea.	Double blind, Randomized, Placebo-controlled	Infants with acute diarrhea	51	1.75, SD 0.7	Hospital	ORS + *Lactobacillus reuteri* DSM 17938 + Zn, standard ORS	Stool volume, stool consistency, drug administration, hospitalization	No adverse effects. Both groups showed reduction in diarrhea severity.
Ly et al. (33)	USA	Is Na or CHO the main factor in hydration? Will varying the concentrations of each in hydration solutions after exercise promote different levels of fluid retention?	Will an ORS (hypoosmolar, higher Na, lower CHO) rehydrate more over 3.5 h than a sports drink (hyperosmolar, lower Na, higher CHO)?	Double blind, Randomized, Placebo-controlled	Athletes	26	21, SD 3.0	Clinic	H2O, ORS (270 mOsm/kg), sports drink (350 mOsm/kg)	Percent of fluid retained, urine excretion, sodium balance	Reducing sodium imbalance promoted greater rehydration. Both ORS and sports drink had higher fluid retention than water. Fluid retention did not differ between the two experimental treatments, but ORS showed better recovery earlier.
James et al. (45)	UK	Several studies have shown that solutions containing protein after exercise-induced dehydration might offer an advantage in fluid retention over non-protein solutions. No other study specifically isolated the protein component.	Gram for gram, is milk protein more effective than carbohydrate at augmenting fluid retention after exercising in the heat?	Randomized, Counter-balanced, Crossover	Adult males	8	21, SD 3.0	Clinic	CHO drink, CHO + milk protein drink	Cumulative urine output, total fluid retention, net fluid balance	Cumulative urine output was less in protein group. Total fluid retention was greater. Net fluid balance was less negative. Net fluid balance was negative for both trials but only significantly negative in carbohydrate group.
Rowlands et al. (76)	New Zealand	Glycogen stores are depleted during exercise and fluid is lost, decreasing performance.	What osmolality of drink most effectively maintains or recovers athletic performance?	Double-blind Latin-square randomized crossover	Male cyclists	11	29, SD 8	Laboratory	Mizone Rapid (218 mOsmol/kg), PowerAde (281 mOsmol/kg), Gatorade (327 mOsmol/kg), non-caloric placebo	Deuterium oxide enrichment, serum osmolality, gut comfort, peak power	Small increase in deuterium oxide with Mizone Rapid; serum osmolality moderately lower with Mizone Rapid but not significantly; gut comfort highest with Mizone Rapid but only significantly higher than PowerAde; peak power highest with Mizone Rapid but differences inconclusive.
Russo et al. (46)	Italy	A train-low recovery regime may calorically optimize recovery.	Will lower carbohydrate in a milk beverage comprimise recovery outcomes?	Double-blind crossover	Male endurance athletes	8	30 median	Laboratory	Full CHO vs. low CHO dairy milk beverages	Muscle glycogen, body water, gastrointestinal markers	Total fat oxidation greater in CHO. Neither treatment recovered muscle glycogen stores

## 3 Results

### 3.1 Body water homeostasis

Water is the largest single constituent of the body, making up 55–65% of body weight, depending on sex, age, and body fat mass (14). Many chemical reactions and metabolic processes use water as a solvent in the body, and it is the medium through which molecules are delivered to cells and waste products are removed (15). Total body water is roughly 2/3 intracellular fluid (ICF) and 1/3 extracellular fluid (ECF) (16). Three quarters of the ECF is interstitial fluid, while the remainder makes up the blood volume (Figure 1). Interstitial fluid stabilizes the cell environment, normalizing pH and carrying waste products to the lymph. Maintaining the relative volumes of the fluid compartments in the body is crucial to proper tissue function, cardiovascular function, and body temperature regulation (16, 104).

**Figure 1:**
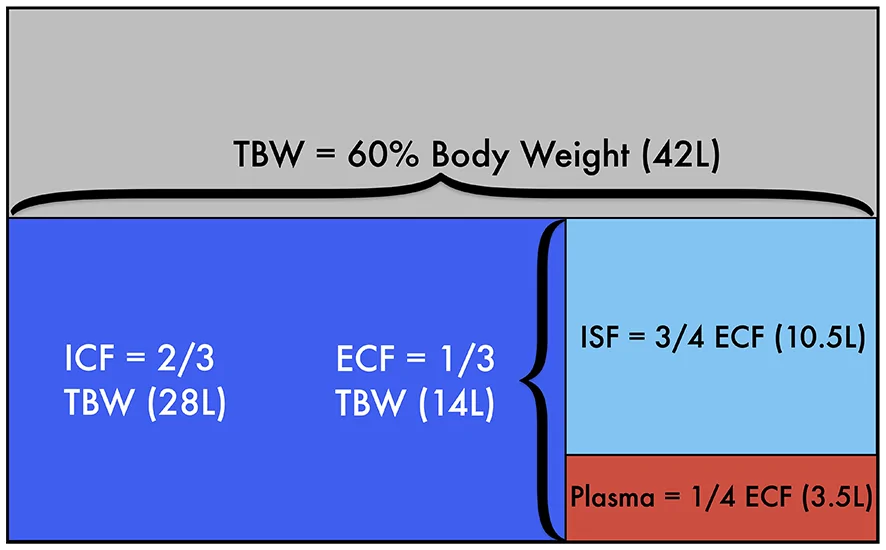
Relative volumes of body fluid compartments. Total area represents a body weight of 70 kg. TBW, Total Body Water; ICF, Intracellular Fluid; ECF, Extracellular Fluid; ISF, Interstitial Fluid. Adapted from Verbalis (14).

Homeostatic fluid balance in the body is maintained by the balance of intake and excretion. Mismatches between fluid intake and loss lead to a body water deficit or surplus. A large component of water flux comes from unregulated sources, such as the water content of ingested food, beverages consumed for secondary content (ex. caffeine, alcohol), sweating, evaporative loss in the breath, gastrointestinal losses, and renal loss necessary to excrete metabolic solutes (14). In normal conditions the body loses approximately 250–350 mL of water per day from respiration and 450 mL per day via diffusion through the skin (6). 100–200 mL per day is lost via the gastrointestinal tract. The greatest avenue of water loss is urine production (1–2 L/d). If these losses are not countered with adequate water intake, the body enters the process of dehydration which results in a hypohydrated state (15).

#### 3.1.1 Measuring biologically relevant concentrations

Plasma concentration is often reported as osmolality, which is the osmolar concentration of all solutes per kilogram of water (15). Osmolality is a colligative property that is independent of the types of solutes present, and is directly measurable by osmometer (17). Plasma osmolality (pOsm) is estimated by adding the concentrations of the main extracellular solutes, Na+, glucose, and urea nitrogen (BUN) (17). Plasma concentration can also be reported in terms of tonicity, which ignores the concentration of solutes that freely pass through cellular membranes, such as small non-polar molecules (N2, O2, CO2), ethanol, steroids, and fat-soluble vitamins. Plasma tonicity is calculated by summing only the osmoles of solutes that do not freely cross cellular membranes, including ions, carbohydrates, amino acids, macromolecules, and hydrophilic molecules (14). Plasma tonicity cannot be measured directly, but is often estimated by subtracting the contribution of membrane-permeable BUN to pOsm (17).

#### 3.1.2 Regulation of plasma volume and tonicity

The kidneys have a primary role in maintaining the volume of the ECF and plasma tonicity (18). Normal pOsm is between 275 and 290 mOsm/kg (15, 18). Plasma osmolality >290 mOsm/kg is detected by hypothalamic osmoreceptors which stimulate arginine-vasopressin (AVP) release and promote thirst (19). An increase in pOsm of more than 2% [[Na+] > 145 mEq/L] results in a doubling of serum vasopressin concentration (20). AVP acts on the principal cells of the collecting duct of the kidney, increasing permeability to water which allows water and solutes to exit the tubular lumen, concentrating urine and lowering serum osmolality (17). Urine osmolality can vary from 50 to 1,200 mOsm/kg depending on the level of sodium and water excretion. Similarly, when a drop in blood pressure occurs, AVP secretion is stimulated by carotid baroreceptors (20).

#### 3.1.3 Water absorption

Water absorption in the human small intestine is a passive process driven by the uptake of effective solutes. Water exits the intestinal lumen either transcellularly through membrane-bound aquaporins, or paracellularly through brush-border membrane vesicles or loosened tight junctions (21). The main mechanism of water absorption in the small intestine is the facilitative intestinal sodium-glucose transporter (SGLT-1). SGLT-1 transports two sodium ions with one glucose molecule, exploiting the osmotic gradient of sodium to transport glucose against its gradient (22). Sodium and glucose absorption are therefore greatly enhanced by the presence of each other (12). The Na+-K+-ATPase pump continuously transports sodium out of the epithelium through the basolateral membrane, creating an electrochemical gradient which facilitates paracellular Cl- absorption (22). Together, the absorption of sodium, glucose, and chloride contribute to the osmotic gradient that facilitates passive water absorption.

### 3.2 Dehydration diagnosis

Dehydration occurs when either an acute or chronic imbalance develops between the homeostatic mechanisms regulating water conservation and excretion. Effective management of dehydration requires understanding the underlying cause, as the same volume of water deficit can produce vastly different biochemical and haemodynamic effects depending on the mechanism of loss (10, 105). In children, dehydration is most often caused by acute gastroenteritis, but can also be caused by other infections such as pneumonia, gingivostomatitis, meningitis, and bronchiolitis (16). Low fluid intake, excessive sweating, burns, cystic fibrosis, vomiting, and diarrhea are other common causes of dehydration (10). Voluntary dehydration is caused by low fluid intake or low consumption of water compared to hyperosmolar fluids like juice and pop.

Dehydration poses several physiological risks, particularly when fluid loss exceeds 3% of the body weight (4). Fluid loss >2% of BW reduces cardiac output, increases perceived exertion during exercise, and impairs cutaneous and thermoregulatory functions (3). Fluid losses exceeding 8% BW can be life threatening, resulting in impaired organ perfusion and oxygenation. Severe acute dehydration (>10% BW) can result in shock, acute kidney injury, coma, and death if not treated immediately (18). Dehydration can impair airway surface liquid distribution in small airways, leading to impaired pulmonary function, though oral rehydration can reverse these effects (23). Dehydration can cause short term memory defects, sensory deficits, mood disturbance, and disruptions in cognition (24). Despite the potentially life-threatening consequences, there are few reliable diagnostic indicators of dehydration.

There are many physical symptoms of hydration, such as long capillary refill time, poor skin turgor, mouth dryness, tachycardia, low systolic blood pressure, dry mucous membranes or axilla, sunken eyes, and decreased saliva flow, but these have poor diagnostic accuracy (15). Decreased bioelectrical impedance, fatigue, and low oral fluid intake usually correlate with dehydration (15). Body mass change compared to a euhydrated baseline is another reliable way to monitor hydration status. Approximately 1mL of water is lost per 1g of body weight loss (6).

Personal cues of hydration status, including level of thirst, urination frequency, and urine color, can also be important for relative assessment, but are not diagnostic in isolation (25). The National Institute for Health and Care Excellence (NICE) recommends using a passive leg lift test to observe any change in haemodynamic condition and uses the following guidelines for evidence of severe hypovolemia: systolic blood pressure < 100 mmHg, heart rate >90 bpm, capillary refill time >2 s (or frigid periphery), and respiration rate >20 breaths per minute (26). Recent methodological recommendations also emphasize that no single hydration biomarker is sufficient to characterize hydration status across all physiological conditions (27). Instead, hydration assessment should integrate complementary measures such as changes in body mass, urine indices, plasma osmolality, and subjective thirst while considering environmental conditions, exercise modality, and individual variability. Urine concentration tests, in particular, should be interpreted with caution, as underhydration (habitually low total body water) may occur without dehydration (an acute reduction in total body water), with both conditions presenting as concentrated urine.

Plasma osmolality is a valuable objective surrogate marker of hypertonic dehydration but there remains a lack of a suitable protocol for direct measurement at the bedside (28). Plasma osmolality can be measured directly with a freezing point depression or vapor pressure depression osmometer, but the cost and lack of trained personnel are often inhibitive to the clinical applicability of these devices. Calculated plasma osmolality (pOsmc) approximates pOsm using the molar concentrations (mmol/L) of the primary osmolytes and the following formula (29):


$$\begin{array}{l} p0sm!=1.86\cdot \left(\left[{Na}^{\prime \prime }\right]+\left[K^{\prime \prime }\right]\right)+1.15\cdot \left[glucose\right]+\left[BUN\right]+14 \end{array}$$

Table 2 reports average healthy values of the primary plasma solutes.

**Table 2:** Mean plasma and intracellular solute concentrations.

Solute	Mol wt.	Valence	Eq wt.	Plasma concentration		ICF concentration
				mmol/L	mEq/L	mEq/L
Na^+^	23	1	23	142	142	14
K^+^	39	1	39	4	4	140
Ca2^+^	40	2	20	2.5	5	4
Mg2^+^	24	2	12	0.85	1.7	35
Cl^−^	35.5	1	35.5	104	104	2
${\text{HCO}}_{3}^{-}$	61	-	22	25	25	8
${\text{H}}_{2}\text{P}{\text{O}}_{4}^{-}{\text{HPO}}_{4}^{2-}$	31	1.8	17	1.3	2.3	40
${\text{SO}}_{4}^{2-}$	32	2	16	0.47	0.94	20
Protein	–	–	–	0.9	15	55
Organic acids	–	–	–	5.5	5.76	68
Glucose	180	–	–	5	5	–
BUN	28	–	–	4.3	4.3	–

Aggregated from (103–105). ICF, Intracellular Fluid; BUN, Blood Urea Nitrogen.

#### 3.2.1 Classification

When available, pOsm measurement or approximation can narrow down the diagnosis of patients presenting with severe dehydration. Plasma hyperosmolality compared to baseline (>300 mOsm/kg) is a likely indication of excess sodium (hypernatremia) relative to TBW, since sodium is the primary extracellular solute (14). Hypernatremia commonly results from blood volume depletion (hypovolemia) due to insufficient water intake. Hypoosmolality (< 280 mOsm/kg) indicates that there is an excess of water relative to an excess of TBW relative to total solutes (or hyponatremia) which results from a combination of water retention and solute loss (15). Since the ECF and the ICF always equalize in osmolality, dehydration resulting in hypernatremia will create an osmotic movement of water from the ICF to the ECF accompanied by intracellular shrinkage. Conversely, hyponatremia shifts water intracellularly, exacerbating a hypovolemic state and stimulating AVP release.

##### 3.2.1.1 Hypertonic dehydration

Hypertonic, low-intake, or water-loss dehydration is defined as an uncompensated TBW deficit, and creates a state of hyperosmolality in the ECF (15). Plasma hyperosmolality can also result from an excess in body sodium, or hypernatremia, but the vast majority of cases are due to losses of body water in excess of body solutes, either by insufficient intake or excessive excretion caused by deficient AVP secretion. Hyperosmolality is almost always synonymous with hypernatremia. A notable exception is diabetic ketoacidosis, where the patient experiences hyperosmolality without hypernatremia (30).

##### 3.2.1.2 Hypotonic dehydration

An excess of water relative to solute in the ECF indicates an overall TBW excess with respect to solutes, representing either a depletion of solute or a dilution from increased TBW (14). A state of hypoosmolality often involves both solute depletion and water retention, but in hypoosmolar patients presenting with dehydration, solute loss profoundly outweighs the volume of water depletion. Hypotonic dehydration must be treated carefully according to the clinical signs of the patient, and usually given isotonic saline (or hypertonic saline, in the case of hyponatremic encephalopathy) to replace fluid losses while maintaining diuresis (16).

##### 3.2.1.3 Isotonic dehydration

Also known as salt loss dehydration, isotonic dehydration involves the loss of both water and salts proportionately. An osmotic differential between the ECF and ICF does not develop, with negligible extracellular redistribution of fluid and AVP release (16). Isotonic dehydration is frequently caused by diuretic use or secretory diarrhea. A blunted AVP response intravascular fluid losses are greater than in hypertonic dehydration because of Verbalis (14). Intravascular fluid loss of more than 10% stimulates baroreceptors in the arteries which promote fluid retention, thirst, and vascular changes through the renin-angiotensin-aldosterone system, AVP, and sympathetic nervous system engagement.

### 3.3 Formulation of oral rehydration solutions

#### 3.3.1 Osmolality

Water and electrolyte absorption from ORS intake primarily depends on the osmotic gradient in the intestinal lumen. Drinking plain water in a state of hypohydration or electrolyte imbalance causes a drop in plasma osmolality which attenuates thirst and promotes diuresis before fluid losses are fully restored (31). Hypotonic and isotonic rehydration solutions contain effective solutes that buffer acute changes in pOsm and create an osmotic gradient favorable to water uptake. Ingestion of hypertonic solutions while dehydrated creates an osmotic pull into the intestinal lumen, ultimately resulting in net efflux of water from the body (3). Hypertonic beverages also reduce the rate of gastric emptying, creating a bottleneck for rapid absorption (3).

In general, intestinal water absorption is inversely correlated with osmolality, with hypotonic solutions producing greater water absorption than iso- or hypertonic solutions (21). Beverages < 270 mOsmol/L aid water absorption by promoting a favorable osmotic gradient to evacuation of water from the small intestine across the mucosa (3). A study in rats showed that hypotonic ORS created less sodium secretion and more potassium absorption in the small intestine than commercially available isotonic solutions, suggesting that hypotonic solutions may promote greater water retention under these experimental conditions (32). In athletes, hypotonic ORS was shown to promote greater hydration than a hypertonic sports drink during early recovery from exercise (33). Collectively, the available evidence supports that hypotonic ORS are important for promoting increased hydration in both animals and humans (32, 33). That said there are few investigations that have directly compared multiple ORS formulations under standardized clinical conditions and therefore future studies are needed to identify optimal osmolality for current ORS formulations.

#### 3.3.2 Composition

A fluid's rate of ingestion depends on the rate of gastric emptying, intestinal fluid absorption, fluid redistribution and fluid retention (3). Palatability, viscidity, and organoleptic properties influence rate of consumption and ingestion, especially at longer timescales (6). For a rehydration formula to optimize the rate of ingestion it must be suitable to the class of dehydration and the source of fluid losses. For example, losses from diarrhea more frequently lead to hyponatremia, so a higher salt content is needed to replace the losses than what would be required for rehydrating following thermoregulatory sweating (10). Beverages formulated primarily for fluid replacement can also be used as a vehicle for other nutrients which may provide clinical benefits in certain cases. The addition and concentration of each component must be analyzed separately and considered based on the purpose of the hydration solution. Common additives are carbohydrate for energy, caffeine for alertness, and protein for muscle recovery.

#### 3.3.3 Sports beverages

Hypertonic (330–730 mOsm/L) sports beverages such as Gatorade^®^ and Powerade^®^ has been shown to increase overall fluid losses when consumed post-exercise, and are not suitable for treatment of secretory diarrhea or severe dehydration (11). Commercial sport drinks are formulated for short-term recovery and provision of energy, and thus contain more carbohydrate relative to electrolytes (3). In contrast, ORS are formulated to replace fluid and electrolyte losses following either acute or chronic hypohydration. Compared to the standard WHO 2002 ORS (245 mOsm/L), sports drinks have higher carbohydrate, lower electrolyte content, more additives, and higher osmolality.

#### 3.3.4 Carbohydrate

Carbohydrate influences the rate of water absorption because of the cotransport of glucose and sodium across the intestinal mucosa. A minimum of 0.9% glucose concentration is needed to enhance water absorption through the gastrointestinal route (6). Ingestion of carbohydrate maintains blood glucose concentration and rate of carbohydrate oxidation, replacing depleted stores that may be low due to physical activity (34).

Since carbohydrates are transported across the intestinal mucosa with different transporters, their absorption rates vary, but multiple carbohydrate sources in the same solution enhances solute and water absorption because of multiple independent forms of intestinal transport (21). For example, when glucose and fructose are ingested together, intestinal absorption of carbohydrate increases, as well as sodium and water (6). Glucose and galactose are co-transported with sodium across the intestinal mucosa via SGLT-1. Fructose is transported by GLUT5 through facilitated diffusion and is absorbed faster than passively absorbed carbohydrates. Fructose is emptied the quickest from the stomach, followed by galactose, followed by glucose, suggesting that the addition of fructose and galactose may deliver nutrients to the small intestine faster than glucose alone (3). The addition of sucrose to a glucose-electrolyte solution was found to increase total carbohydrate absorption in rat intestinal perfusion (32). However, other studies have found very similar rates of uptake from disaccharide solutions compared to equimolar monosaccharide solutions (7). There is sufficient evidence to suggest that sodium absorption is enhanced by the presence of multiple carbohydrates in a solution (21).

##### 3.3.4.1 Starch

The type of glucose found in an ingested solution also influences sodium absorption. Sodium absorption is the greatest alongside glucose polymers, followed by maltose, followed by sucrose (21). High glucose concentration (>111 mmol/L) increases the osmotic load of the solution which may further aggravate fluid loss and induce hypernatremia (35). In addition, glucose contained in maltose, maltotriose, oligosaccharides, and polymers are absorbed faster than free glucose, likely due to increased local concentration around transport sites following enzymatic hydrolysis on the brush border (21). To introduce more carbohydrate without the additional osmotic load, complex carbohydrates from fruits, vegetables, rice, wheat, sorghum, and maize have been incorporated into ORS formulations (6). Starches such as amylopectin, maltodextrin, and high amylase-resistant starch have lower osmolality than mono- and di-saccharides, and release glucose slowly as it is slowly broken down in the small intestine (6). Glucose polymers also increase gastric emptying (3).

Incorporating starches may have the downside of decreasing palatability (36). Most of the evidence supporting specific polymers is based on physiological mechanisms or selected formulations rather than direct clinical comparisons with standard glucose-based ORS. Therefore, although starch-based ORS may offer advantages by providing carbohydrates without substantially increasing osmolality, current evidence does not establish if starch-based ORS consistently improves rehydration outcomes compared with conventional glucose-based ORS.

#### 3.3.5 Protein

Protein is often included in fluid replacement beverages to aid in muscle protein synthesis following strenuous exercise, though its influence on rehydration when added to ORS is small and may depend on the specific amino acids (37). Gastric emptying is not influenced by the type and concentration of amino acids that occur in beverages (3). Amino acids are absorbed using independent sodium- coupled transporters which can create an additive effect to sodium absorption (38). In piglets with rotavirus enteritis, the addition of glutamine to ORS improved sodium chloride absorption (39). However, in human proximal jejunostomy patients the addition of glutamine to ORS did not increase fluid or sodium absorption (40). It is noted that glutamine and other nitrogen-containing compounds can negatively influence palatability of the solution.

The benefits of consuming protein are identical when consumed 0–2 h pre- or immediately post- exercise (41). During exercise, intake of 0.25 g/kg of protein per hour may alleviate muscle soreness and lower creatine kinase levels (42). A drink formulated with protein may aid in muscle recovery when taken before, during, or after strenuous exercise, as long as the level of protein consumption does not exceed the absorptive rate or cause gastric upset. A clinical trial with thirteen participants showed that a carbohydrate-based drink with protein added had 15% more retention than a carbohydrate alone. The drinks were consumed during a 3 h rehydration period following 2.5% body weight dehydration through cycling (38). Branched chain amino acids (BCAAs), particularly leucine, have been identified as an important dietary component of muscle recovery, decreasing creatine kinase and muscle soreness post-exercise (43). Both short term and long term BCAA loading regimes have decreased indirect markers of muscle damage. A 2017 study in resistance-trained athletes found that BCAA supplementation improved post-exercise strength and soreness (44). Limited data is available on the effectiveness of BCAA supplementation during exercise or as an ORS additive. Therefore, although protein may provide additional recovery benefits during exercise, there is insufficient evidence to conclude that protein or specific amino acids consistently enhance ORS-mediated rehydration.

#### 3.3.6 Milk

Apart from carbohydrate and protein, dairy milk contains a suite of lipid molecules that also impact absorptive rates upon ingestion (37). Milk does not increase muscle glycogen stores, but milk protein might aid in fluid retention (45, 46). Milk during recovery was shown to decrease urine output following exercise in a warm environment compared to a sports drink and water (47). Liquid dairy has a higher leucine content than other protein sources, and may also provide higher bioavailability of essential amino acids (48). The lipid content in milk makes a low-carbohydrate milk formulation particularly promising in keto-adapted endurance training regimes.

#### 3.3.7 Micronutrients

##### 3.3.7.1 Sodium

Sodium is the primary extracellular osmolyte. Sodium is also the primary electrolyte in sweat, making it a priority for replacement following exercise (31). Ingestion of sodium and water maintains interstitial and plasma volume. An inverse relationship has been observed between urine output and sodium concentration in rehydration drinks (37). Higher sodium concentrations in rehydration drinks are associated with greater fluid retention, but impact palatability and compliance, creating a barrier to patient acceptance of ORT (11).

##### 3.3.7.2 Potassium

Potassium is the primary cation in the intracellular compartment. A proper balance of sodium and potassium has an established physiological role in neural transmission, muscle contraction, and smooth muscle tone (6). Potassium is commonly included in oral rehydration beverages to replace losses through sweating or diarrhea (49). A KCl drink consumed *ad libitum* was more successful in recovering intracellular fluid volume compared to an NaCl drink in rats dehydrated by 9% of body weight (50). However, emerging evidence as to whether higher potassium concentration enhances rehydration remains conflicting, but the data supports potassium being a beneficial cation for inclusion in ORS formulations (37). Potassium does not significantly contribute to solute absorption at the concentration found in rehydration drinks (21).

##### 3.3.7.3 Chloride

Chloride is the major anion in the extracellular space, and usually has equal osmolar concentration to sodium from the dissolution of NaCl (37). Chloride is lost during sweating through the osmotic pull of sodium loss (6). There is a close relationship between sodium and chloride absorption in the small intestine and colon, and those ions that are not absorbed in the small intestine are effectively reclaimed in the colon (32). There is little evidence that chloride itself should be an additive to ORS, but it contributes to the overall solvent drag which creates a favorable osmotic gradient for water absorption.

##### 3.3.7.4 Zinc

Zinc is a trace mineral which is involved in maintaining integrity of the gut lining (51). A zinc fortified oral rehydration solution improved intestinal permeability and mucosal recovery in children with gastroenteritis (52). In the case of treating diarrhea, replenishing micro nutrients such as zinc can be an important component of rehydration therapy. In children with acute diarrhea, zinc supplementation was shown to decrease symptoms by 12–25%, though it is unclear whether this is due to a replenishment of zinc loss or a protective effect of zinc on water retention (53). Compared to a weight-matched zinc control, zinc-deficient rats were shown to have decreased absorption capacity and higher fluid and electrolyte losses when stimulated by *Vibrio cholerae* enterotoxin (51). Zinc was shown to enhance Na absorption and inhibit Cl secretion in rat ileal mucosa (54).

Overall, the available evidence suggests that zinc supplementation may be beneficial in the management of acute diarrhea, although its specific role in rehydration is still unclear. Differences between clinical and experimental studies make it difficult to determine whether zinc directly improves fluid absorption or whether its benefits are mainly related to intestinal recovery. While zinc may therefore be useful as a component of ORS for diarrheal illness, there is currently limited evidence to support whether its addition to ORS formulation is beneficial for improving rehydration in other settings.

##### 3.3.7.5 Magnesium

Magnesium plays key roles in muscle contraction, bone development, vascular tone, cardiac excitability, and neuron function (55). Recommended daily intake is between 300 and 400 mg, though only about a third of dietary intake is absorbed. Magnesium supplementation may improve systolic blood pressure during recovery from exercise by up to 12 mmHg, but the effect is dependent on dietary intake (56). Acute loading with magnesium provided a performance benefit during bench press, while chronic loading provided no significant benefit (56). Emerging evidence suggests that magnesium may be considered as an ORS additive in the context of exercise, but its effectiveness may be negligible if the level of dietary intake is already high.

Magnesium can cause osmotic diarrhea if ingested at high concentrations, but at physiological levels it was shown to decrease Cl- secretion in an *in-vitro* cholera mouse intestine model through inhibition of the Ca2+-sensing receptor (57). Including the replenishment of Mg2+ lost in diarrhea or through sweat, magnesium may be a beneficial additive to ORS due to its enhancement of fluid retention in models of secretory diarrhea.

#### 3.3.8 Bases

Base compounds are added to rehydration solutions to normalize the pH and reduce instability. Bicarbonate, acetate, and citrate promote absorption of sodium and water in the healthy intestine, contradicting findings that a slightly acidic pH enhances water and sodium absorption as well as preserves shelf-life and palatability (3, 49). Bases have an overall neutral effect on water absorption, and except in the case of metabolic acidosis, ORS without added base is equally effective (58).

##### 3.3.8.1 Bicarbonate

In 1985 sodium bicarbonate was substituted for sodium citrate in the WHO-ORS formulation because of its longer shelf life and reduced packaging costs (11). Bicarbonate has demonstrated efficacy in enhancing exercise performance, particularly during 1–10 min bursts of high intensity effort (59). Elevated blood bicarbonate has an established role in pH stabilization both intracellularly and extracellularly, which can enhance the transport of H+ and lactate from the muscle into the circulation (60). There have been inconsistent results among studies that have examined bicarbonate supplementation during exercise, but many report a positive effect on muscle endurance, anaerobic capacity, and peak anaerobic power (61). Emerging evidence suggests an ORS designed for acute bicarbonate loading prior to short high intensity exercise may improve exercise performance in healthy athletes. A US study in 97 children found that low serum bicarbonate (< 17 mmol/L) indicated moderate to severe dehydration, suggesting that supplementation of bicarbonate in an ORS would offset a deficit incurred by dehydration (62).

Collectively, studies on bicarbonate supplementation have produced mixed results, with some reporting benefits for high-intensity exercise and others show little or no performance benefits. These differences may reflect variations in the dose and timing of bicarbonate supplementation, as well as differences in exercise protocols and study populations. Bicarbonate therefore may be useful for buffering during high-intensity exercise or correcting acid-base disturbances. That said whether it provides an additional benefit for rehydration when included in ORS remains unclear.

##### 3.3.8.2 Pyruvate

Emerging preclinical evidence has demonstrated that pyruvate is a promising alternative to other base molecules in fluid formulations. In animal studies it has been shown to be more effective than acetate, bicarbonate, chloride, lactate, and malate in resuscitation fluids for shock treatment (63). In mice with severe injuries, intestinal absorption of water and sodium was increased by 20% with pyruvate ORS compared to standard WHO-ORS (64). Pyruvate ORS also improved histopathological alterations. In hemorrhagic shock models, enteric rehydration with pyruvate ORS prolonged survival, corrected lactic acid acidosis, prevented fatal systemic oxidative stress and inflammation, and increased visceral blood flow compared to bicarbonate (WHO-ORS) (63). The clinical use of pyruvate in ORT has yet to be demonstrated outside of animal models.

#### 3.3.9 Other compounds

Various natural products and additives have been added to rehydration solutions for energy provision or other secondary health benefits, but their effects on water retention and gut mobility are not well-studied. Diet is a factor that can be managed alongside ORT to promote water retention. Diets that are high in fiber and promote gut health such as the Mediterranean diet may improve nutrient and liquid absorption (65). Green banana and pectin were shown to decrease vomiting, diarrheal duration and recovery period as well as need for oral rehydration and intravenous fluid in children with persistent diarrhea (66).

##### 3.3.9.1 Caffeine

Caffeine is used in fluid formulations to aid focus and concentration, particularly in energy drinks. Caffeine in large doses (250–300 mg) has a profound diuretic effect on those without a recent history of consumption, but minimal effect on those that consume coffee or tea regularly (67). Doses found in standard servings of coffee, tea, and soft drinks do not have a significant diuretic effect, and thus have a neutral effect on hydration. Consuming large doses in an ORS formulation may induce net water loss in patients naive to caffeine.

##### 3.3.9.2 Short chain fatty acids

Short chain fatty acids (SCFA) such as butyrate are the primary stool anion but are not present in the diet (12). SCFA are synthesized by colonic bacteria from non-absorbed carbohydrates through fermentation. SCFA are rapidly absorbed in the colon and stimulate fluid and Na absorption, thus the colon becomes a site for reclaiming carbohydrate, calories, fluid, and electrolytes. SCFA can be delivered in the form of resistant starch to avoid amylase digestion in the small intestine (2). ORS formulated with high amylose maize starch showed a 30–50% reduction in time to first formed stool two studies with adults (2).

##### 3.3.9.3 Ketones

Ketones are produced by the oxidation of fatty acids in the liver during periods of hypoglycemia. They function as an alternative energy substrate for peripheral tissues when glucose needs to be spared for brain and neural functioning (68). Ketones can attenuate glucose metabolism in the periphery and regulate muscle readiness as a metabolite (69). Overall metabolic energy extraction may improve under acute nutritional ketosis achieved by supplementation with exogenous ketone esters (70). A 2025 study showed no difference in exercise performance following a recovery period where endurance-trained males drank a recovery beverage enhanced with ketone esters, though the ketone treatment lowered glycemia and increased carbohydrate retention (71). Consumption of ketone esters before and during a shuttle run decreased plasma lactate but did not provide a marked change in performance (72).

There is a lack of evidence whether ketone bodies are a suitable additive to ORS. A Spanish observational study found that ketoanemia is not a predictor of ORS failure in dehydrate pediatric patients with vomiting, suggesting that supplementing ketones would not necessarily influence ORS efficacy (73). Given the profound effect of ketones on glucose metabolism, they should be avoided in solutions intended for recovery and maintenance.

##### 3.3.9.4 Gum arabic

Gum arabic is a soluble fiber obtained from the Acacia senegal tree. In male juvenile rates, gum Arabic enhanced net water and sodium absorption in the jejunum when added to ORS (74). In a model of chronic osmotic-secretory diarrhea, gum arabic enhanced ORS increased potassium, sodium, and water absorption by 2-fold (74).

### 3.4 Exercise rehydration

Effective rehydration during exercise and post-exercise recovery requires a balance of fluid and electrolytes. A body water deficit equivalent to as little as 2% of body weight may impair performance, especially if starting exercise in a hypohydrated state (37).

#### 3.4.1 During exercise

Drinking too much or too little fluid may be detrimental to exercise performance. Hydration should be structured when exercising in repeated sessions with short recovery time, as thirst-driven (*ad libitum*) fluid intake is not sufficient to restore a euhydrated state in the short term (36). Drinking >150% of body weight loss during exercise can result in hyponatremia, especially during ultra distance events (75).

Hypotonic drinks have been shown to promote greater rehydration than iso- and hypertonic drinks during exercise. As exercise duration increases, hypotonic drinks have demonstrated greater efficacy while isotonic drinks deteriorate in their capacity to hydrate (3). A hypotonic drink ingested at regular intervals during exercise can effectively promote rehydration. Although sodium is reabsorbed in the duct of the sweat gland, sodium secretion increases proportionally to sweating rate, suggesting that a higher sodium content may be necessary for maintaining electrolyte balance during intense exercise (6).

The most substantial impact from a rehydration solution during exercise may come from the composition of carbohydrates. Rowlands et al. (76) found no difference in cycling performance when comparing the hydration of hypo-, iso-, and hypertonic drinks. However, maximizing carbohydrate oxidation during long distance (>2.5 h) events requires a carbohydrate intake of up to 90 g/h (33). SGLT-1 absorption becomes saturated at 60 g/h, which supports the use of multiple transportable carbohydrate sources in in-event hydration fluid (21).

#### 3.4.2 Post-exercise

In a hypohydrated state following exercise, the body will continue to lose water through the obligatory excretion pathways, so a greater volume than what was lost must be consumed to restore fluid balance. Ingesting 150% of body weight loss after strenuous exercise, particularly in the heat, has shown to recover hydration effectively (37). Replenishment of electrolytes, particularly sodium, is paramount to exercise recovery (47). Ingestion of a concentration of sodium equal or greater to that lost through sweat is required to achieve positive fluid balance, and indeed, urine output is inversely proportional to sodium intake following exercise (37). Sufficiently high concentrations of carbohydrate in a hypotonic recovery drink improves water retention. Protein (particularly milk or high-BCAA mixtures) aids in muscle protein synthesis in the hours immediately following exercise so should be considered as a post-exercise additive. Many other micronutrients are lost or displaced during exercise in small amounts, but there is little evidence of their supplementation expediting exercise recovery. Recent recommendations also emphasize rehydration strategies during exercise should be tailored to the individual athlete according to exercise duration and intensity, environmental conditions, and individual athlete characteristics, as all influence the degree of dehydration that occurs (27).

### 3.5 Oral hydration therapy in healthcare

Dehydration is associated with various diseases, pathological conditions, chronic conditions, and demographics. Vulnerable demographics include performance athletes, the elderly, and hospital patients in critically ill conditions. In Europe, 20% of those surveyed in Germany, Spain, and Greece were dehydrated (4). In African countries, 80–90% of children are dehydrated. In the United States, dehydration ranges from 16 to 28%, those in older demographics being more susceptible. Individuals greater than 60 years of age are particularly susceptible to hypohydration. As we age, thirst response is dampened by reduced renin-angiotensin-aldosterone activity, baroreceptors decrease in sensitivity, and the number of functioning nephrons decreases, impairing urine concentrating ability (77).

The presence of dehydration within healthcare systems is a major concern due to poor diagnosis and detection, as well as the known association with increased mortality and co-morbidities (15). In 2000, Commonwealth Fund report found that the degree to which dehydration has reached prevalence in nursing homes can be described as a “silent epidemic” (78). In hospitalized elderly patients, dehydration is associated with increased length of stay, readmission and economic burden (15).

Comorbidities are often the cause of dehydration, but other causes include aging, polypharmacy, hot weather, and limited access to drinking water. Hypertension and diabetes mellitus both have a component of renal impairment, from which dehydration results, either from the disease itself or from pharmacological diuretics. Patients with kidney conditions resulting in impaired urinary concentrating ability are at high risk for volume depletion and dehydration (16). If the kidney has an inability to reabsorb solutes, this results in excessive urinary fluid losses and leads to dehydration.

#### 3.5.1 Clinical applications

Oral Rehydration Therapy has been introduced in clinical settings as an alternative to intravenous fluids (IVF) (79). Children with acute diarrhea are effectively treated with ORS to restore electrolyte balance and promote reabsorption of water (16). Rice-based ORS have been particularly effective in reducing stool volume and diarrheal frequency (80, 81). ORT has been successful in replacing up to 77.5% of IVF requirements in treating intense burns within the first 24 h (82). ORT is used as a hydration supplement before surgery to reduce stress and anxiety associated with preoperative fasting, as well as improve postoperative insulin resistance and haemodynamic balance (83, 84). Oral fluid intake is regulated by homeostatic mechanisms, posing less risk of fluid overload and subsequent edema (82). ORT was highly preferred and more cost effective than IV or protracted infusion delivery of chemotherapy in metastatic colorectal cancer patients (9).

#### 3.5.2 Long term care

Patients in long term care facilities are at particularly high risk of dehydration. A UK study found that one in five patients in long term care are dehydrated, and half are at risk of becoming so (15). These patients are typically senior, which alone is a risk factor for impaired fluid balance and poor kidney function (77). Seniors are also more frequently on a course of medication that may include laxatives and diuretics, which can promote dehydration. The most effective therapeutic strategy has been to adjust standards of care to incorporate better fluid intake regimens, more frequent documentation of intake, and more drink availability (85). These measures can be as simple as verbal reminders to drink more fluids (86). ORS has been shown to recover mild dehydration in seniors over a 5 day period (86).

#### 3.5.3 Diabetes insipidus

Diabetes insipidus (DI) results from either low AVP secretion or renal desensitization to AVP and causes pure water diuresis due to a lack of urine concentration ability (14). DI can be central, caused by a defect in the hypothalamus-pituitary axis, or nephrogenic, caused by a defect in the AVP V2 receptor. Polyuria (excessive urination) and polydipsia (excessive thirst) are common, and hypernatremia will develop if there is insufficient access to water. If a patient is hospitalized and presents with hypernatremia, IVF administration with 5% dextrose solution is appropriate. Management of heritable DI could also represent an emerging application of ORS, with fluid losses being measured frequently and fluid prescription being adjusted accordingly (17).

#### 3.5.4 Diabetes mellitus

Diabetes mellitus is characterized by abnormally high blood glucose levels (hyperglycemia), either due to a lack of insulin production (Type 1) or an acquired resistance to insulin (Type 2). A prolonged state of hyperglycemia can lead to many complications, including kidney disease, nerve damage, ocular damage, and vascular ischemic events (87). Severe acute hyperglycemia can lead to increased hepatic ketogenesis and metabolic acidosis, resulting in severe dehydration, hypovolemia and osmotic diuresis (88). These cases require treatment in the ICU, as they can be life threatening and lead to coma or death. IVF is usually necessary to restore blood volume, first with isotonic saline, then hypotonic saline as plasma osmolality stabilizes. ORT has not been considered in these critical situations.

Patients with managed diabetes are still at increased risk of dehydration because of osmotic diuresis and altered fluid reabsorption in the kidney (89). Diabetics may be more prone to dehydration due to certain lifestyle factors such as fluid intake, alcohol consumption, and strenuous exercise. This risk is exacerbated by high temperatures, diarrhea, acute illnesses, fever, nausea, and vomiting (89). Certain antidiabetic medications may also exacerbate electrolyte imbalance and fluid retention abnormalities. ORT represents an emerging potential intervention to mitigate these risks, especially during times of stress, illness, and excessive heat.

#### 3.5.5 Inflammatory bowel disease

Inflammatory Bowel Disease (IBD) is a chronic immune-related inflammatory disease of the gastrointestinal tract. It is a multisystem condition, also affecting the musculoskeletal, ocular, and cutaneous systems (90). Diet and hydration are critical components of IBD management. A diet rich in plant fiber, fresh fruits and vegetables, monounsaturated fats, complex carbohydrates, and lean proteins, is strongly recommended (91). The Mediterranean diet in particular has demonstrated improved microbiome diversity, cardiovascular risk, and metabolic syndrome risk in the general population. Case studies with Mediterranean diet intervention have achieved symptomatic remission in IBD patients (65). Internal strictures may prevent patients from consuming fibrous foods, so care must be taken to cook and process foods into a soft texture for easier digestion. Intestinal damage limits the absorption capability of the intestinal tract, potentially to the point where liquid nutrition may need to be provided through a feeding tube (enteral nutrition, EN) or intravenously (parenteral nutrition, PN) (91). Beverages with a high osmotic load should be avoided, and hypotonic ORS are recommended to improve intestinal absorption. PN can lead to bloodstream infections, thrombotic complications, and hepatobiliary complications, and therefore direct oral hydration with ORS is preferred (91).

Chronic malabsorption, dietary restriction, and mucosal inflammation often leads to malnutrition in IBD patients. Patients are frequently deficient in vitamin D, iron, and vitamin B12. Depending on other risk factors, patients can also be deficient in zinc, copper, folic acid, and vitamins A, E, and K (91). Of these, the inclusion of B12 and zinc as additives to an ORS formulated for IBD represents an emerging formulation strategy.

#### 3.5.6 Crohn's disease

Characterized by relapsing-remitting episodes of intestinal inflammation, Crohn's disease is a chronic disorder causing significant morbidity and mortality. It can affect any part of the gastrointestinal tract (90). One in five patients are hospitalized during the course of disease, while 12.4% of patients must undergo surgery, either due to inflammatory flare-ups or non-inflammatory complications (92). Hospitalization rates have not significantly decreased in the last two decades despite advances in therapeutic modalities. Symptoms include diarrhea, constipation, weight loss, abdominal tenderness, fistula, stricture, and extra-intestinal complications. Patients with chronic inflammation and diarrhea are at particular risk of dehydration. Best-practice recommendations for IBD are also applicable to Crohn's, but there are no studies specifically investigating rehydration regimes in Crohn's patients. The Crohn's disease exclusion diet consists of whole foods that limit changes to the microbiome and intestinal barrier. In combination with a hydration regime, as well as separating liquid intake from solid food at mealtimes, dietary changes may most effectively remediate malabsorption and encourage intestinal adaptation.

#### 3.5.7 Short bowel syndrome

About 8L of fluid enters the small intestine each day, and most is recovered by the ileum and colon (93). Short bowel syndrome (SBS) is a condition that arises following surgical resection of the ileum or colon due to congenital defects or disease. SBS is characterized by poor nutrient absorption and loss of electrolytes including zinc, copper, and magnesium (94). SBS patients are at high risk of diarrhea, dehydration, and electrolyte imbalance (95). SBS carries a risk of intestinal failure which requires intravenous fluid (IVF) or parenteral nutrition (PN) to maintain nutrient and fluid balance (8).

ORT has been successful as a replacement or partial substitution for IV therapy and PN in jejunostomy patients managing dehydration at home (8). ORT has been shown to intravenous fluid requirements in patients with SBS who require PN (79). If patients are weaned onto ORS while in hospital it will be easier to continue at home, and will be less painful and easier to adjust to than IV or subcutaneous saline. Substitution with ORS can curtail the effects on the patient of IV or PN supply shortages. If ORS is not available at home, the recommendation is to use diluted sports drinks or diluted fruit juice, though these options will likely have lower sodium concentration than is required to enhance reabsorption (8). If the patient has a partial colon, liquids may not be well-tolerated, but ORS that are formulated to rebalance electrolytes may be provided (94).

Consuming fluid in a large bolus creates gastric dumping, which should be avoided in patients with SBS (93). Sipping a glucose based electrolyte solution was shown to most effectively recover salt loss in patients with high output jejunostomy, compared to salt capsules and the glucose polymer solution Maxijul (96). A regimen of drinking 1L of solution in small amounts throughout the day is the most effective method of maintaining euhydration, though patients without a colon may need 2–3L of ORS a day (8). No more than four ounces of fluid should be consumed at once. This method prevents edema, renal failure, and excess stoma in patients on oral fluid restriction during recovery from jejunostomy.

During long term rehydration, an isotonic solution is recommended to prevent fluctuations in serum osmolality (97). Formulating isotonic solutions with maltodextrins and a high sodium content may increase sodium and water absorption (98). Hypertonic fluids promote influx of sodium and water into the gut lumen and should be avoided. Similarly, drinking plain water should be avoided as it leads to diuresis and net sodium loss. At least 90 mmol/L of sodium is recommended, with higher salt contents increasing absorption but sacrificing palatability. Oral supplementation of sodium is preferable to sodium tablets, as these can cause vomiting in some patients (96). Patients may develop hypomagnesemia, which should be counteracted with magnesium supplements as opposed to being an additive in oral hydration (97). Similarly, for the purposes of chronic use, potassium chloride may be excluded from the ORS. Sodium bicarbonate may also be excluded and replaced with sodium citrate to enhance palatability. Glucose-polymer solutions or a rice-based solution can increase the energy intake without increasing the osmolality (96).

### 3.6 Oral rehydration therapy alternatives

#### 3.6.1 Probiotics

The gut microbiome is critical for gut health and aids in nutrient digestion and absorption. Probiotics protect from gastrointestinal disease by improving intestinal barrier function, but the effects also come from modifications to immune responses (99). Enrichment of ORS with probiotics may improve hydration outcomes. A randomized controlled trial in patients with acute diarrhea found no difference in stool output between standard ORS and ORS enriched with zinc and *Lactobacillus reuteri*. The enriched ORS was well-tolerated with no adverse effects (100). Treatment of children with cystic fibrosis using *Lactobacillus GG* resulted in fewer pulmonary exacerbations and hospital admissions compared to ORS (99).

#### 3.6.2 Medications

There are a number of medications that assist in hydration or boost rehydration potential, to be used in patients with a short bowel or severe, non-responsive high-volume stoma. Loperamide and codeine phosphate, the former being recommended as it is not addictive, are antimotility drugs that decrease ileostomy sodium and water output by 20–30% (97). Loperamide operates in the entero-hepatic circulation which is disrupted in jejunostomy patients and a very high dose may be required, but these doses are associated with serious cardiac events (97). Drugs that reduce gastric secretions may also be effective in patients with stomal output >2L/24 h.

Protein pump inhibitors and H2 antagonists inhibit enzymatic secretion of gastric acid and reduce hypersecretion. They do not have any effect on absorption of energy, carbohydrate, lipids, nitrogen or divalent cations. Proton pump inhibitors are not routinely used as they have a range of side effects including myocardial infarction, renal impairment, and osteoporosis (97).

### 3.7 New technologies in hydration

A comprehensive review of industry hydration developments is beyond the scope of this article, but a selection of current products is provided here to demonstrate emerging hydration innovations. A majority of recent advancements remain unpublished, and there is limited data supporting the efficacy of proprietary formulas in improving electrolyte and water absorption. Both published and unpublished investigations have focused on optimizing performance and carbohydrate delivery during strenuous exercise. There remains a knowledge gap with regards to how these more recent technologies may be efficacious in managing chronic conditions or treating acute dehydration as part of an ORT regime.

Gastric emptying is delayed with increasing carbohydrate concentration and osmolality, which can cause gastrointestinal stress during high-intensity exercise (76). Hydrogels containing carboxylic acid-rich polymers change structure depending on the pH environment (101). Hydrogel-based carbohydrate delivery systems encapsulate carbohydrates using an alginate and pectin matrix which dissolves in the small intestine. These formulations are proposed to increase gastrointestinal tolerance and allow higher carbohydrate consumption per hour without provoking gastrointestinal distress, though clinical evidence remains limited. The encapsulated maltodextrin-fructose mixture has been proposed to allow higher carbohydrate concentrations without a delay in gastric emptying.

Hydrogel formulations have also been investigated as a potential delivery system for bicarbonate supplementation to reduce pH changes from lactate accumulation which is commonly associated with gastric upset typically experienced when bicarbonate is ingested. However, evidence supporting this application is currently limited, and further independent studies are needed to determine its effects on hydration and exercise performance. Toney (101) tested the Maurten hydrogel against a standard maltodextrine-fructose solution and a maltodextrin only solution. Cyclists experienced performance benefits with the hydrogel and maltodextrine-fructose solution compared to maltodextrin only, but there were no differences in gastrointestinal self-reporting throughout the trial. Importantly, these studies evaluated exercise performance rather than hydration efficacy. The effects of a hydrogel formulation on electrolyte and water absorption have not been studied.

Glycogen contains branched glucose chains that are easily metabolized by the liver, but not in the intestine. One example is cyclic cluster dextrin (CCD), a highly branched cyclic polysaccharide. CCD resembles the branched-chain structure of amylopectin. These properties may facilitate carbohydrate delivery during prolonged exercise, although supporting evidence remains limited. CCD is more soluble than glycogen and amylopectin, however, the physiological significance of this difference for gastric emptying and gastrointestinal tolerance has not been clearly established. Currently evidence supporting CCD has focused primarily on carbohydrate metabolism and exercise performance rather than hydration outcomes. Further research is needed to determine whether CCD-containing beverages improve fluid retention compared with conventional ORS formulations, as current peer-reviewed evidence remains insufficient.

## 4 Discussion

ORS is the most accessible form of acute hydration and remains one of the most life preserving medical developments in the 20th century. Since the 1970s it has become a component of treatment in a variety of electrolyte and fluid balance disorders. Consuming ORS effectively promotes positive fluid balance by inducing sodium-glucose co-transport and providing a favorable osmotic gradient for absorption. ORS is preferable for hydration purposes in high-intensity and high-temperature environments, including endurance sports, high altitude, spaceflight, and military activities. Any condition which increases fluid loss through sweat, urination, or stoma can potentially include an ORT regime.

Even during acute dehydration from burns, ketoacidosis, or polyuria, ORT can be an effective alternative to IVF for restoring fluid and electrolyte levels in selected clinical settings. When fluid replacement is required long-term, IVF and PN can impair patient livelihood and incur excessive costs, whereas ORS can be prepared at home and can be self-administered.

Hypotonic ORS formulations induce an osmotic gradient that favors absorption, but the precise osmolality that achieves optimal fluid balance in various chronic conditions has not been investigated. Most clinical trials have only compared a single ORS formulation against other beverages, and few have compared multiple commercially available ORS to each other. Isotonic solutions may be preferable to hypotonic solutions when fluid absorption is compromised long-term.

Glucose and sodium are the essential components to ORS, but many other additives and formulations exist that change the absorptive and organoleptic properties. Higher sodium concentration is beneficial to absorption and reduces urine output but negatively impacts palatability and adherence. Other electrolytes have an unclear influence on fluid retention, but zinc and magnesium may be beneficial for maintaining gut integrity and reducing negative ion secretion. Adding milk protein or glutamine to ORS may cooperatively promote sodium absorption but results are inconclusive in human patients. Formulations with soluble fibers from rice, maltodextrins, SCFAs, or gum Arabic reduce the osmotic load and utilize ileal and colonic absorption. Ketone esters offer an alternative energy source to carbohydrates that could potentially maximize energy extraction from an ORS.

Hydration also has implications for long-term health. In athletes, habitually low water intake has been associated with increased renal concentrating activity as well as the activation of fluid regulatory mechanisms, even when plasma osmolality and total body water are within the normal range (102). In non-athlete populations, chronic low water intake has also been associated with changes in renal and metabolic health, though it is currently unknown if athletes also experience these long-term effects (102).

Researching whether athletes are vulnerable to these effects is particularly relevant as they repeatedly experience fluid loss through training and competitions. Therefore, to ensure optimal health, hydration strategies should also consider the possible effects of hydration habits over time instead of just recovery from acute dehydration. Further longitudinal studies are needed to determine whether habitual hydration practices influence renal, metabolic, and cardiovascular health both during an athlete's competitive career and afterwards.

Investigations into the composition and efficacy of rehydration solutions were primarily carried out more than two decades ago, and despite continued advances in hydration physiology, relatively little innovation has been translated into clinically validated ORS formulations. In contrast, the exercise rehydration industry has recently developed several new technologies primarily focus on efficient carbohydrate delivery. These advances provide strong rationale to suggest that these new technologies may have a clinical application to improve hydration outcomes in clinical patients in the hospital and at home. However, important questions remain regarding their efficacy, safety, and applicability across different disease states. Current ORS technologies are effective for treating acute dehydration but may not optimally target the distinct physiological mechanisms that underlie chronic disorders. Additionally, many of these emerging technologies are supported primarily by mechanistic studies, pre-clinical investigations, or a limited number of clinical trials. Therefore, future research should prioritize independent randomized clinical trials comparing novel formulations with the established ORS, standardized assessments of hydration outcomes and the development of disease specific ORS formulations as there are distinct physiological mechanisms involved with different disorders. In addition, because investigations of these technologies have focused primarily on exercise performance rather than water and electrolyte absorption, future studies should evaluate both performance and hydration-specific outcomes, including fluid retention, electrolyte replacement, restoration of plasma volume, and recovery of hydration status, to determine whether these novel substrates and technologies can be successfully translated into oral rehydration therapy.

Beyond improving patient care in developed healthcare systems, advances in ORS formulation may also have important humanitarian applications. Powdered ORS formulations are inexpensive, lightweight, and have a long shelf life, which makes them well-suited for disaster relief in areas with limited access to clean water. Therefore, improvements in formulation that enhance water and electrolyte absorption while maintaining stability, affordability, and its ease of transport would increase the effectiveness of oral rehydration programs delivered by organizations such as the World Health Organization (WHO) and other humanitarian agencies. Future research should therefore consider both the physiological effects of novel ORS formulations, as well as their scalability, cost-effectiveness, and suitability for deployment in environments where access to intravenous therapy, medical infrastructure, and potable water may be limited. Successfully translating advances in hydration science into stable, evidence-based powdered ORS formulations have the potential to improve outcomes for millions of individuals affected by humanitarian crises.
